# Neck Grasp Predicts Obstructive Sleep Apnea in Type 2 Diabetes Mellitus

**DOI:** 10.1155/2019/3184382

**Published:** 2019-07-01

**Authors:** P. J. Edmonds, K. Gunasekaran, L. C. Edmonds

**Affiliations:** ^1^Vanderbilt University Medical Center, Department of Medicine, 1161 21st Avenue South, Nashville, TN 37232, USA; ^2^Yale New Haven Health, Bridgeport Hospital, Pulmonary Medicine, 267 Grant Street, Bridgeport, CT 06610, USA; ^3^Sleep Disorder Center, Research Institute, Bassett Medical Center, 1 Atwell Road, Cooperstown, NY, USA

## Abstract

**Aims:**

Obstructive sleep apnea (OSA) is a common disorder with high morbidity, mortality, and an increasing prevalence in the general population. It has an even higher prevalence among individuals with type 2 diabetes mellitus (DM). The snoring, tiredness, observed apnea, high blood pressure, body-mass-index, age, neck circumference and male gender (STOP-BANG) questionnaire and Berlin Questionnaire can be cumbersome in clinical practice and require subjective data on sleepiness. We proposed prospectively studying a primary care population with type 2 DM comparing neck grasp, neck circumference, and common screening questionnaires to identify OSA.

**Methods:**

Persons with a diagnosis of type 2 DM were recruited from a primary care clinic. Participants were screened using Easy Sleep Apnea Predictor (ESAP), STOP-Bang questionnaire, and Berlin questionnaire. A positive ESAP was defined as a 1cm gap when a patient encircled their hands around the neck. All subjects underwent in-laboratory PSG testing.

**Results:**

Forty-three participants were enrolled and the prevalence of OSA was 90.7% (AHI ≥ 5). The median BMI was 38.0. The prevalence of mild OSA by PSG (AHI 5-14) was 27.9%, moderate OSA (AHI 15-29) was 25.6%, and severe OSA (AHI >30) was 37.2%. For mild OSA both ESAP and neck circumference showed 100% specificity.

**Conclusions:**

This study reinforces the need for screening diabetic persons for obstructive sleep apnea. ESAP and neck circumference are useful for identifying persons with type 2 DM who are at risk for OSA. Together these findings could improve recognition of OSA in persons at risk for cardiovascular disease. Trial Registration of “Neck grasp as a predictor of Sleep Apnea,” https://clinicaltrials.gov/ct2/show/NCT02474823, Clinical Trials.gov Identifier, is NCT02474823.

## 1. Introduction

Obstructive sleep apnea (OSA) is a common disorder with high morbidity, mortality, and increasing prevalence [1]. Moreover, despite efforts to improve recognition, 80% remains undiagnosed [2]. In the general population obstructive sleep apnea affects 3-7% of persons [3]. Persons with type 2 diabetes mellitus (DM) are at increased risk for OSA with a reported prevalence ranging from 23-90% [4–6]. OSA is an independent risk factor for type 2 DM [7]. Both OSA and type 2 DM are strong risk factors for cardiovascular disease [3, 8–10].

While several questionnaires and models have been validated for the clinical prediction of sleep apnea, few have been validated in a population of type 2 diabetes mellitus [11–13]. The snoring, tiredness, observed apnea, high blood pressure, body-mass-index, age, neck circumference and male gender (STOP-Bang) questionnaire and Berlin Questionnaire (BQ) [14] can be cumbersome in clinical practice and require subjective data on sleepiness and third party reporting of snoring [11, 14]. A simple, reliable, and objective screening test could improve a timely diagnosis of OSA in persons with type 2 DM.

Neck circumference has a well-studied relationship with OSA. Decreased cross sectional area, increased compliance, and mass loading of the upper airway all contribute to sleep disordered breathing [15]. Guidelines suggest screening for OSA with a collar size greater than 17 inches in males and 16 inches in females [16]. A previous study of neck grasp (easy sleep apnea predictor, ESAP) for identifying mild OSA (AHI>5) reported a sensitivity of 68.3% and a specificity of 100% in a symptomatic sleep clinic population [17].

We proposed to prospectively study the usefulness of the simple neck grasp, neck circumference, BMI (body mass index) and common screening questionnaires to identify OSA in type 2 diabetes mellitus. We hypothesized that a positive ESAP is noninferior to Stop-Bang, Berlin Questionnaire at recognizing OSA in type 2 DM.

## 2. Methods

**Figure 1:**
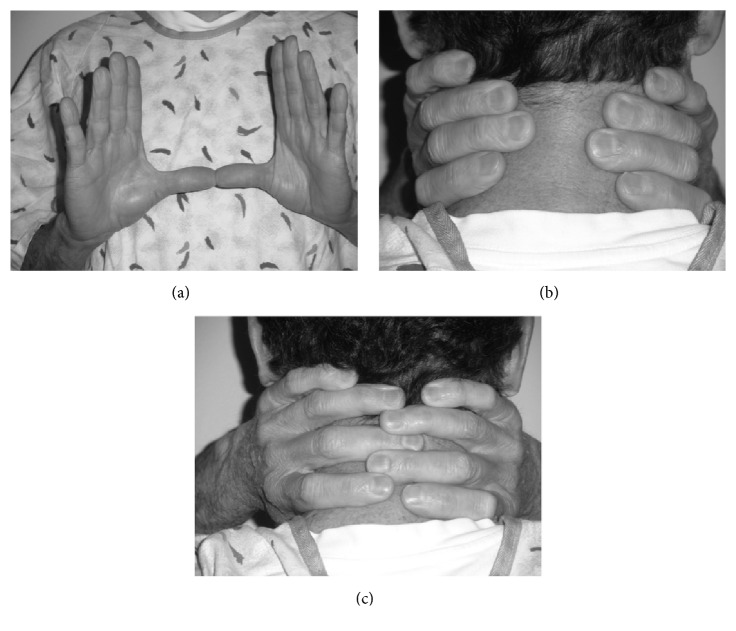
(a) Easy sleep apnea predictor (ESAP) entails placing both thumbs together at the anterior of the neck and encircling the fingers in the posterior. (b) Positive ESAP is shown. The patient is unable to encircle their neck resulting in at least a 1cm gap between fingertips. (c) Negative ESAP is shown. The patient is able to encircle their neck defined as touching fingertips in the posterior neck. Figures reproduced with author permission [17].

The institutional review board approved the study design involving noninvasive procedure with human subjects. The approved protocol (IRB number 2012) included informed signed consent from all participants and oversight to ensure operation within the approved protocol. Adults (age 18 years and older) with a diagnosis of type 2 DM were serially recruited from a general internal medicine clinic at routine health visits from 2015-2016. Exclusion criteria included a known diagnosis of OSA, age <18, unstable cardiopulmonary disease, inability to perform the neck grasp or complete questionnaires. Participants were screened using the ESAP (easy sleep apnea predictor), STOP-BANG questionnaire, neck circumference (>16 inches in females and > 17inches in males) [16] and Berlin questionnaire. A positive ESAP was defined as a 1cm gap when patient encircled their hands around the neck (see Figure 1). All subjects underwent in-laboratory Type 1 polysomnogram (PSG). Type 1 polysomnogram was scored using AASM 2016 scoring rules, specifically hypopnea rule of 3% or arousal. Participants were reimbursed 50 dollars for participation.

### 2.1. Statistical Analysis

Demographic variables between groups were compared using Chi-squared and the t test (BMI, age, AHI). Data are presented as mean ± standard deviation. Using AHI from polysomnogram as the gold standard for OSA diagnosis the sensitivity (SN), specificity (SP), positive predictive value (PPV), and negative predictive value (NPV) to predict OSA were calculated. Sample size calculation used prevalence of OSA (7% in the general population and estimated 20% in diabetes mellitus) type 1 error (0.05) and power (0.8). Continuous variables (BMI, neck circumference, and ESAP gap measure) were compared to AHI using the receiver operator curve to calculate sensitivity (SN), specificity (SP), and area under the curve (AUC). An AUC of ≥0.9 indicated outstanding discrimination, 0.8 ≤ AUC < 0.9 indicated good discrimination, 0.7 ≤ ACU <0.8 indicated acceptable discrimination, and AUC =0.5 indicated no discrimination.

## 3. Results

### 3.1. Study Population Characteristics

Eight-six participants were screened in a primary care clinic. Forty-three persons met inclusion criteria (53% female, Table 1). The most common reasons for exclusion were prior diagnosis of OSA or not having diabetes mellitus. The prevalence of OSA was 90.7%. The median BMI was 38.0. The prevalence of mild OSA by PSG (AHI 5-14) was 27.9%, moderate OSA (AHI 15-29) was 25.6%, and severe OSA (AHI >30) was 37.2%. In the study population 11.63% of the subjects were uncertain if they snored. The neck circumference was increased (>43.2 cm in males and >40.6 cm in females) in 44% of the participants. As expected in a population of type 2 DM, the BMI was ≥35 in a majority of subjects (65.12%).

### 3.2. Validation of Screening Tests for Mild, Moderate, and Severe OSA

For Mild OSA (AHI 5-14) the most sensitive screening test was STOP-Bang score ≥ 3 with a sensitivity of 87.2%. The most specific screening tests were ESAP and neck circumference both with 100% specificity (Table 2). For moderate OSA (AHI 15-29) the most sensitive screening test was the Berlin high-risk group. The most specific screening test was neck circumference with a specificity of 75% (Table 2). The most sensitive screening tests for Severe OSA (AHI≥ 30) were STOP-Bang and the Berlin Questionnaire both with a sensitivity of 93.8%. The most specific screening test was neck circumference with a specificity of 70.4% (Table 2).

### 3.3. Linear Regression of Continuous Variables

Continuous variables (BMI, neck circumference, and ESAP gap measure) were compared to AHI (Table 3). In participants with a positive ESAP, the number of centimeters between the fingers was used as a gap measure. Area under the recover operator curve was highest for neck circumference (0.885) followed by ESAP gap measure (0.724), and BMI (0.705) (Table 3). STOP-Bang and BQ were not included because they were scored dichotomously.

## 4. Discussion

In this study, we demonstrate that neck circumference performs well as a clinical screening tool in the diabetic population. Furthermore, we show that the ESAP neck grasp is a simple alternative to measuring neck circumference. Participants with type 2 DM have a high prevalence of obstructive sleep apnea, which is consistent with prior reports [4–6].

Our key findings are the usefulness of neck girth based predictors (neck circumference and ESAP) of OSA in type 2 DM. With the high specificity and positive predictive value, ESAP is reliable at recognizing mild disease (AHI≥5). The sensitivity, specificity and area under the curve for neck circumference and ESAP showed similar results (100% specificity for mild OSA), with neck circumference showing slight superiority. One advantage of ESAP compared to neck circumference is that gender normative standards need not be applied (>17 inches in males and >16 inches in females) [16]. Hand to neck size is individually normative. The lower sensitivity of neck circumference and ESAP compared to STOP-Bang and BQ questionnaires could be attributed to craniofacial abnormalities, tonsillar or adenoidal enlargement not assessed by neck girth alone. Unlike STOP-BANG and the Berlin Questionnaire, ESAP requires no other information or point assignment to predict OSA (AHI≥5) in type 2 diabetics. In addition to simplicity, relying on neck girth avoids subjective questioning. Snoring status, an important component of STOP-Bang and BQ, was uncertain in 11.6% of study participants.

We confirmed prior findings that neck circumference is a better predictor than BMI alone [18] and that guidelines using neck circumference remain useful [19–21]. STOP-BANG has similar sensitivity and specificity compared to prior studies in surgical and primary care patients [11, 14, 22]. Berlin Questionnaire also showed similar sensitivity and specificity to prior validation [23]. A negative ESAP or a normal neck size does not exclude OSA because of the low sensitivity. However, a positive test correlates well with persons who are at risk for OSA.

As continuous variables, ESAP and neck circumference did not reliably predict severity of OSA, nor did BMI. As a noncontinuous variable BMI also lacked the specificity seen in neck girth based tests. The neck-based predictors likely see the increase specificity over BMI due to anatomical role of mass loading on the upper airway in OSA.

The relationship between OSA and type 2 DM was originally attributed to obesity, but further research has shown OSA is an independent risk factor for type 2 DM [7]. OSA affects physiologic and hormonal pathways including hypoxia induced changes in glucose metabolism [24], sleep fragmentation affecting hypothalamic-pituitary-adrenal axis [25], and increased levels of IL-6, TNF-alpha, adiponectin, and MCP-1 [26–28]. Numerous studies show an association between sleep apnea and cardiovascular disease [29], glucose metabolism [30], and systemic hypertension [31]. OSA is an independent risk factor for all-cause mortality after controlling for obesity and cardiovascular disease [3, 8–10]. Guidelines from the International Diabetes Federation and the American Diabetes Association recommend screening all individuals with type 2 DM for OSA [19, 32].

The strengths of this study include using a prospective study design that enrolled participants who were not presenting with sleep complaints. By using in lab polysomnography instead of HST as the gold standard false negatives or miss classifications are minimized. In addition to simplicity, relying on neck girth avoids subjective questioning. Snoring status, an important component of STOP-Bang and BQ, was uncertain in 11.6% of study participants.

Limitations of this study include the small sample size and the higher than anticipated prevalence of OSA in the population. Areas of further research include testing if our findings would be useful in other similarly obese populations, such as bariatric surgery patients. Neck grasp is unstudied in truck drivers and department of transport examinations, a population where it might be useful to avoid historical bias against reporting snoring and sleepiness.

In summary, this study reinforces the importance of screening for obstructive sleep apnea in persons with type 2 DM. ESAP and neck circumference are useful for identifying individuals with DM2 who are at risk for mild OSA. A negative ESAP or normal neck circumference does not exclude OSA. Together these findings could improve recognition of OSA in individuals at risk for cardiovascular disease.

## Figures and Tables

**Table 1:** Patient demographics and characteristics.

Characteristic	N (%)
Male	20 (46.51)

Female	23 (53.49

AHI^†^ group (by PSG)	

Positive (AHI>5)	39 (90.7)

Mild (AHI 5-14)	12 (27.91)

Moderate (AHI 15-29	11 (25.58)

Severe (AHI≥30)	16 (37.21)

BMI^§^ ≤35	15 (34.88)

BMI>35	28 (65.12)

Positive Neck Circumference*∗*	19 (44.19)

Negative Neck Circumference	24 (55.81)

*Mean (*+/- *SD, range)*	

AHI	31.24 (SD +/-28.14, 1-117)

BMI (kg/m^2^)	38.26 (SD +/-7.69, 24-55)

Neck Circumference (cm)	41.64 (SD +/-4.04, 33-51)

Gap measure^¶^ (cm; ESAP negative = 0 cm)	3.20 (SD +/-3.81, 0-13)

† AHI (apnea-hypopnea index is the number of apneas and hypopneas per hour of recording. This was scored using type 1 polysomnogram with AASM 2016 scoring rules, specifically hypopnea rule of 3% or arousal.

§ BMI (body mass index) is the weight in kilograms divided by the square of the height in meters.

¶ Gap measure was the number of cm between the fingers in a patient with a positive ESAP.

^*∗*^A positive neck circumference was >16 inches in females and > 17inches in males. A negative neck circumference was <16 inches in women and < 17 inches in men.

**Table 2:** Comparison of for screening tests for sensitivity, specificity, positive predictive value, and negative predictive value for mild OSA, moderate, and severe OSA.

Mild OSA (AHI 5-14, n= 12)				

Screening Test	Sensitivity	Specificity	Positive Predictive Value	Negative Predictive Value

Positive ESAP*∗*	55	100	100	18.2

STOP-Bang ≥ 3	87.2	0	89.5	0

Berlin High Risk^†^	79.5	0	88.6	0

BMI ≥ 35	69.2	75	96.4	20

Positive Neck Circumference^§^	48.7	100	100	16.7

Moderate OSA (AHI 15-29, n=11)				

Positive ESAP	60.7	68.8	77.3	50

STOP-Bang ≥ 3	92.6	18.8	65.8	60

Berlin High Risk	88.9	31.3	68.6	62.5

BMI ≥ 35	74.1	50	71.4	53.3

Positive Neck Circumference	55.6	75	78.9	50

Severe OSA (AHI >30, n=16)				

Positive ESAP	62.5	57.1	45.5	72.7

Stop-Bang ≥ 3	93.8	14.8	39.5	80

Berlin High Risk	93.8	25.9	42.9	87.5

BMI ≥ 35	75	40.7	42.9	73.3

Neck Circumference	68.8	70.4	57.9	79.2

*∗*A positive ESAP (easy sleep apnea predictor) is defined as the failure to encircle the neck using hand grasp with a 1cm gap between fingers (Figure 1).

^†^High-risk berlin questionnaire is 2 or more positive categories on the screening questionnaire.

^§^A positive neck circumference was >16 inches in females and > 17 inches in males.

**Table 3:** Comparison of Linear regression (c-statistic) for ESAP, BMI, neck circumference, and gap measure.

Screening Test	Area under the curve
ESAP	0.657

BMI (continuous)	0.705

Neck Circumference (cm)	0.885

Gap Measure*∗* (cm)	0.724

^*∗*^Gap measure was the number of cm between the fingers in a patient with a positive ESAP. STOP-Bang and BQ were not included because they were scored dichotomously.

## Data Availability

The data used to support the findings of this study are available from the corresponding author upon request.
